# Gut Microbiota and the Quality of Oral Anticoagulation in Vitamin K Antagonists Users: A Review of Potential Implications

**DOI:** 10.3390/jcm10040715

**Published:** 2021-02-11

**Authors:** Anny Camelo-Castillo, José Miguel Rivera-Caravaca, Esteban Orenes-Piñero, Inmaculada Ramírez-Macías, Vanessa Roldán, Gregory Y. H. Lip, Francisco Marín

**Affiliations:** 1 Department of Cardiology, Hospital Clínico Universitario Virgen de la Arrixaca, University of Murcia, Instituto Murciano de Investigación Biosanitaria (IMIB-Arrixaca), CIBERCV, 30120 Murcia, Spain; anjo134@gmail.com (A.C.-C.); jmrivera429@gmail.com (J.M.R.-C.); minmaculadarm@gmail.com (I.R.-M.); 2 Liverpool Centre for Cardiovascular Science, University of Liverpool and Liverpool Heart and Chest Hospital, Liverpool L7 8TX, UK; gregory.lip@liverpool.ac.uk; 3 Department of Biochemistry and Molecular Biology-A, University of Murcia, Instituto Murciano de Investigación Biosanitaria (IMIB-Arrixaca), CIBERCV, 30120 Murcia, Spain; eorenes@um.es; 4 Department of Hematology and Clinical Oncology, Hospital General Universitario Morales Meseguer, University of Murcia, 30008 Murcia, Spain; vroldans@gmail.com; 5 Department of Clinical Medicine, Aalborg Thrombosis Research Unit, Aalborg University, 9000 Aalborg, Denmark

**Keywords:** oral anticoagulants, gut microbiota, microbial metabolites, vitamin k, trimethylamine n-oxide

## Abstract

The efficacy and safety of vitamin K antagonists (VKAs) as oral anticoagulants (OACs) depend on the quality of anticoagulation control, as reflected by the mean time in therapeutic range (TTR). Several factors may be involved in poor TTR such as comorbidities, high inter-individual variability, interacting drugs, and non-adherence. Recent studies suggest that gut microbiota (GM) plays an important role in the pathogenesis of cardiovascular diseases, but the effect of the GM on anticoagulation control with VKAs is unknown. In the present review article, we propose different mechanisms by which the GM could have an impact on the quality of anticoagulation control in patients taking VKA therapy. We suggest that the potential effects of GM may be mediated first, by an indirect effect of metabolites produced by GM in the availability of VKAs drugs; second, by an effect of vitamin K-producing bacteria; and finally, by the structural modification of the molecules of VKAs. Future research will help confirm these hypotheses and may suggest profiles of bacterial signatures or microbial metabolites, to be used as biomarkers to predict the quality of anticoagulation. This could lead to the design of intervention strategies modulating gut microbiota, for example, by using probiotics.

## 1. Introduction

For many years, the only options available for oral anticoagulation (OAC) therapy were the vitamin K antagonists (VKAs), and they are still widely used for several conditions. The most common indication is stroke prevention in atrial fibrillation (AF) [1], so the majority of AF patients require long-term OAC, as suggested by guidelines [2,3]. Despite the introduction of direct-acting oral anticoagulants (DOACs), the use of VKAs in AF is common in some countries, for example in Eastern Europe and Southern Europe [4]. Aside from AF patients, there is also a wide range of patients for whom VKAs are the recommended OAC therapy, for example, AF patients with rheumatic mitral stenosis and the presence of mechanical valvular prostheses [3,5]. In these patients, evidence about the safety and efficacy of DOACs is limited, and VKAs are the most commonly used therapy, thus highlighting the need for good quality anticoagulation control in these VKA users [6].

Similarly, in patients with history of thrombosis and diagnosed with antiphospholipid syndrome (APS), the use of rivaroxaban was associated with an increased risk of recurrent thrombotic events, compared with warfarin [7]. In the absence of evidence for apixaban, edoxaban, and dabigatran, all DOACs are not recommended in patients with APS, particularly in high-risk patients (those who present a positive test for all 3 antiphospholipid tests: lupus anticoagulant, anticardiolipin antibodies, and anti-beta 2 glycoprotein I antibodies). In addition, APS patients taking a DOAC should be carefully evaluated and the possibility of switching to a VKA should be considered. Thus, VKA therapy remains the first-line treatment for a first or recurrent APS-related venous thrombotic event [8]. Finally, DOACs are currently more expensive than treatment with VKA and in the context of venous thromboembolism (VTE), there is no reimbursement for DOACs in several countries [9,10]. Consequently, an important proportion of VTE patients who could be potential users of DOACs are actually taking VKAs.

In patients who are prescribed VKAs, the quality of anticoagulation control is central to prevent bleeding and thromboembolic events [11]. There is evidence for gut microbiota influencing the progression or development of some cardiovascular diseases [12,13], but there are limited data linking gut microbiota to the quality of anticoagulation in VKA users. The aim of the present review is to summarize published data on the potential impact of the gut microbiota on the quality of anticoagulation of patients receiving VKA therapy, and to suggest different hypotheses that could associate intestinal bacterial with VKA.

## 2. Main Text

### 2.1. Search Strategy and Selection Criteria

Data for this review were identified by searches of PubMed, and references from relevant articles using the search terms “oral anticoagulants,” “vitamin K antagonists,” “gut microbiota,” “microbial metabolites,” “vitamin K,” and “trimethylamine N-oxide.” Articles published in English and Spanish between1980 and 2020 were reviewed and included, if appropriate.

### 2.2. Quality of Anticoagulation with VKA

Underuse and premature discontinuation of OAC is common, and VKAs have a major inter- and intra-individual variability with a narrow therapeutic window [14]. Also, regular monitoring is required to maintain the International Normalized Ratio (INR) between 2.0 and 3.0 (therapeutic range), to ensure a time in therapeutic range (TTR) ≥65–70% [3,15].

Nevertheless, half of VKAs users have poor anticoagulation control [16], resulting in an increased risk of thromboembolic and bleeding events, including ischemic stroke, major bleeding, major adverse cardiovascular events (MACEs), and all-cause (and cardiovascular) mortality [11]. Several factors may predispose to poor TTR, including interacting drugs, non-adherence to therapy, and concomitant conditions [17] (Table 1), as well as dietary factors.

In addition, VKAs inter-individual variability is often so high that it is difficult to clarify why TTR decreases or why an optimal TTR is never achieved. Therefore, one possibility may be due to diet and the influence of gut microbiota.

### 2.3. Gut Microbiota and Xenobiotic Metabolism

The human microbiome is composed of a consortium of bacteria, viruses, archaea, eukaryotic microbes and their genomes in dynamic and symbiotic equilibrium with host cells [18,19]. This ecological community of symbiotic, commensal, and pathogenic microorganisms has a potential role contributing to health and disease [20]. The microbiota is known to impact human physiology, protect against pathogens, contribute energy homeostasis, neurodevelopment, extract nutrients from the diet [21], metabolism, and normal immune functions [19,22].

Microbial cells that colonize the human body, including skin and mucous membranes, are as abundant as our somatic cells, being estimated between 500 and 1000 species of bacteria. The densest habitat in the human body is the gut, with high microbial biomass (0.15 kg) with the *Firmicutes* and *Bacteroidetes* as dominant phyla [23]. The human microbiome is highly personalized, with a unique composition in each individual and some factors can profoundly affect the structure of the microbial community, such as diet, lifestyle, and antibiotics. Moreover, the human microbiome is highly dynamic and its composition can vary over short-term and long-term timescales. In early life, for example, the establishment and composition of gut microbiota during infancy is affected by the type of infant feeding, birth mode, older siblings, and maternal or infant antibiotic use [24]; subsequently, after the first year of life, the gut microbiota increases dramatically in diversity and stability.

Microorganisms modify many classes of dietary components, such as lipids, proteins, polysaccharides, phytochemical complexes or small molecules (xenobiotics) [25]. They also transform industrial chemicals, altering their toxicity and lifetimes in the body. As for drugs, the bacteria can change their pharmacokinetics and pharmacodynamics, affecting critically the activation of the prodrug, its biological effect and half-life, as well as bioavailability, resulting in adverse effects or loss of effectiveness. Most human microbiota-xenobiotic interactions are performed in the gastrointestinal tract, where there are regions with different pH, oxygen levels, cellular physiology, and amounts of nutrients [26].

The combined metabolisms of host and microbiota can produce metabolites that may alter the lifetime and bioactivity of xenobiotics in the human body [27,28]. At the enzymatic level, the host mainly uses oxidative and conjugative chemical reactions. Human xenobiotic metabolism often transforms non-polar compounds into hydrophilic metabolites that are more easily excreted, a process that occurs in two phases [29].

The xenobiotic compounds that are ingested orally pass into the small intestine, suffering enzyme-mediated modifications and being absorbed by host tissues. Before xenobiotics are transported to the liver through the portal vein, they pass through or between intestinal epithelial cells, undergoing enzymatic processes [30]. After the exposure to liver enzymes, xenobiotics and their metabolites pass into the systemic circulation, being distributed through tissues and distal organs. In the circulatory system, they are excreted or metabolized, either by biliary excretion or through the kidneys. Metabolites that returned to the light of the intestine are excreted with the feces or can be reabsorbed by enterohepatic circulation and transformed by gut bacteria [31,32]. Compounds administered by other pathways, for example by intravenous injection or easily absorbed compounds, can be also exposed to the action of gut microorganisms [33]. At the same time, metabolites produced by intestinal microorganisms can circulate systemically [34], interacting with epithelial cells of the gastrointestinal tract locally or be absorbed by the host and eliminated by feces or urine [35], generating a complex metabolic network that affects both, the host and the microbiota itself. Within the distinctive and complex ecology of the human gut, microorganisms transform ingested substrates through a wide range of enzymatic reactions, with inter-individual variability in the ability to transform or metabolize xenobiotics [36]. Despite the consequences of these modifications, little is known about the individual microbes and enzymes involved in these reactions.

**Figure 1:**
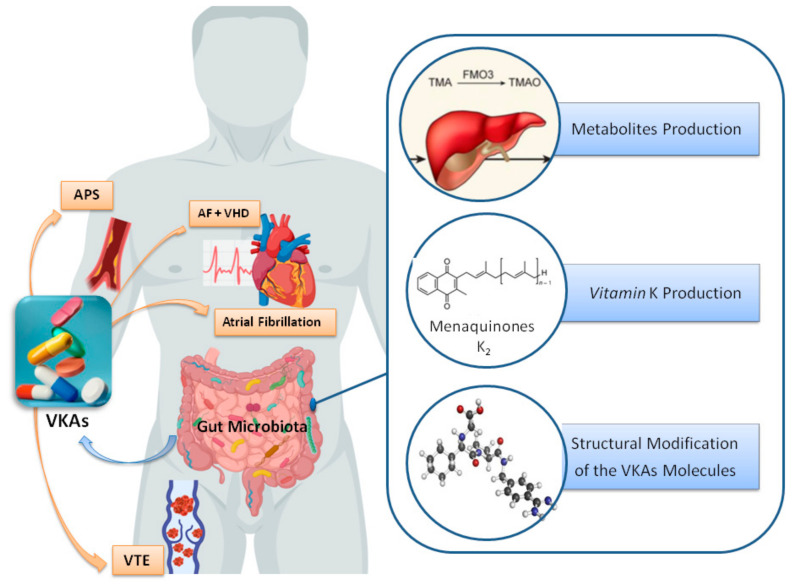
Potential impact of the gut microbiota in vitamin K antagonists. Three hypotheses about the role of the gut microbiota on the quality of anticoagulation in patients taking vitamin K antagonist therapy: (i) Effect of microbial metabolites in the bioavailability of VKAs; (ii) bacterial production of vitamin K and; (iii) structural modification of VKA molecules. Abbreviations: AF, atrial fibrillation; APS, antiphospholipid syndrome; VHD, valvular heart disease; VKAs, vitamin K antagonists, VTE, venous thromboembolism.

Given the above evidence, we explore three different hypotheses about the potential impact of the gut microbiota on the quality of anticoagulation in patients receiving anticoagulant therapy with VKAs. First, an indirect effect of metabolites produced by gut microbiota in the availability of VKAs drugs; second, an effect of vitamin K-producing bacteria; and third, structural modification of the VKA drug molecule (Figure 1).

#### 2.3.1. Effect of Metabolites Produced by Gut Microbiota on VKAs Drugs

Gut bacteria generally exert reducing and hydrolytic reactions [37]. Enzymes associated with gut microorganisms are oxydoreductases, hydrolases, transferases, and lyases [38], widely distributed in a variety of bacterial groups, although enzymes with high similarity between families can catalyze different chemical reactions.

The gut microbiome metabolizes non-digestible dietary components [39] and participates in immune function [40] and/or bioactivation of nutrients and vitamins. However, not all the microbial enzymes involved in these chemical transformations have been described. Indeed, not all metabolic activities or functional profiles in these microbial communities can be predicted in metabolomic studies [41]. Thus, metabolic functions are assigned to superfamilies that can catalyze several different chemical reactions. One of the more interesting abilities of intestinal bacteria is the synthesis of metabolites produced directly by the gut microbiota or by the metabolism of dietary components, such as trimethylamine N-oxide (TMAO), indoxyl sulfate (IS), and indole-3 acetic acid (IAA) [42].

##### Trimethylamine N-Oxide (TMAO)

Concerning metabolites, intestinal bacteria metabolize part of the main group of phosphatidylcholine, in the metabolic via phosphatidylcholine-choline, producing an intermediate compound known as trimethylamine (TMA). TMA is generated from dietary compounds such as betaine, L-carnitine, and its metabolite γ-*butyrobetaine* (GBB), choline, and other choline-containing compounds [43].

**Figure 2:**
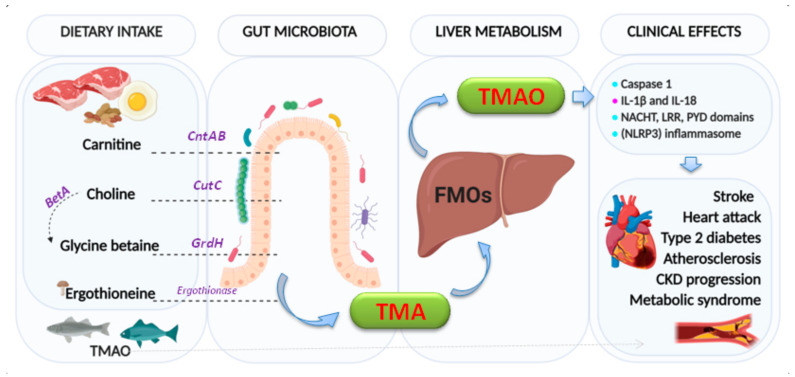
Formation of trimethylamine (TMA) and trimethylamine N-oxide reductase (TMAO). The key enzymes responsible indicated are: *carnitine monooxygenase (CntAB); choline-TMA lyase (CutCD); glycine betaine reductase (GrdH)*; and *Ergothionase.* Choline to glycine betaine is mediated by the Bet pathway (*BetA*). FMOs: flavin-dependent monooxygenase (isoforms 1,3). Clinical effects: 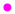: activation, 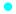: producing and secretion.

Choline is an essential nutrient, contributing to neurotransmission, methyl transfer events, and cell membrane function. It is found in high quantities in food stuffs such as peanuts, eggs, meat, beef liver, and cauliflower [44]. It is obtained as free choline from foods of animal origin or as part of various compounds such as phosphocholine, phosphatidylcholine, and sphingomyelin. Free choline is absorbed throughout the small intestine and it is integrated into cell membranes or absorbed by the liver, where it can be converted to lecithin, phosphocholine, or betaine [45]. If the amount of choline exceeds the absorption capacity, it passes to the large intestine where it is metabolized by microbial action to methylamines [46] (Figure 2).

**Figure 3:**
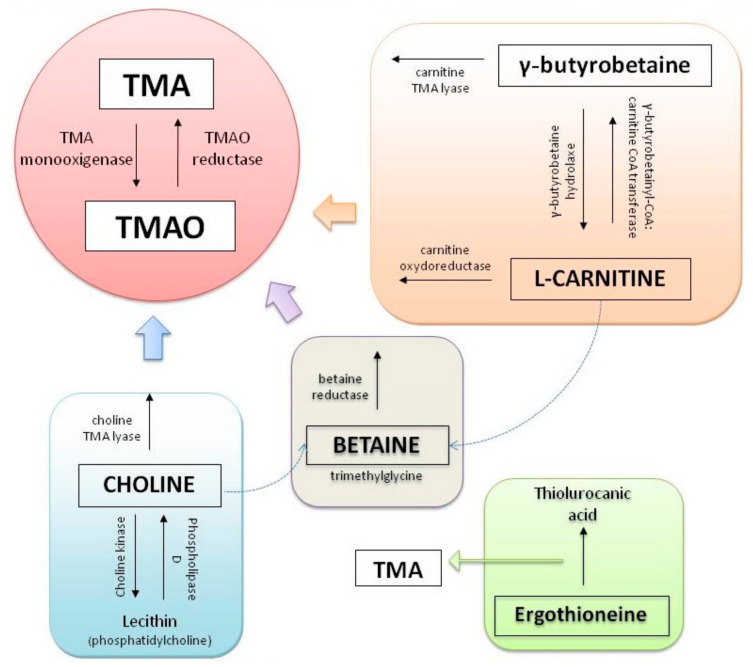
Principal pathways to formation of trimethylamine (TMA) and trimethylamine N-oxide reductase (TMAO).

On the other hand, L-carnitine is converted into TMAO by the action of carnitine oxidoreductase. Next, TMA is rapidly oxidized by hepatic flavin-containing monooxygenases (FMO1–FMO3) to form TMAO [47]. Figure 3 summarizes the principal pathways and enzymes involved in the production of TMA and TMAO.

There is a recognized association between TMAO and inflammation [48,49,50,51,52]. Indeed, TMAO triggers vascular inflammation by activation of caspase 1, producing and secreting pro-inflammatory cytokines (IL-1β and IL-18) and activating the NACHT, LRR, and PYD domains-containing protein 3 (NLRP3) inflammasome [53]. Activation of inflammasome has been linked to heart failure, adverse heart remodeling, and cytokine-mediated systolic dysfunction. The effects of TMAO on the components of the intrinsic cardiac autonomic nervous system (Ganglionated Plexi (GP) [54]) have been associated with increased expression via pro-inflammatory signaling and the nerve growth factors [53]. Whether circulating TMAO derived from the intrinsic microbiome can reach the GPs or not by creating sufficient local concentrations to give rise to arrhythmogenic effects [55] remains to be confirmed.

Likewise, elevated levels of TMAO in plasma increase the risk of developing atherosclerosis [56] and other adverse cardiovascular events, such as stroke, myocardial infarction, and death [47]. Recent studies in primary human coronary artery endothelial cells (HCAECs) isolated from normal human coronary arteries demonstrated that TMAO promoted nuclear translocation of nuclear factor-κB (NF-κB) and expression of tissue factor (TF), implicated in the thrombogenicity of atherosclerotic plaque [57]. Low-dose TMAO also significantly promoted low-dose of tumor necrosis factor-alpha (TNF-α) [58] or high mobility group box 1 (HMGB1) mediated by TF expression via activating NF-κB signaling and finally, studies in ST-elevation myocardial infarction patients, linking increased plasma concentrations TMAO with increased TF activity [59].

##### Indoxyl Sulfate (IS) and Indole-3 Acetic Acid (IAA)

Indoxyl sulfate (IS) is a protein-bound uremic solute resulting from bacterial metabolism of dietary tryptophan to indole by the action of bacterial tryptophanases, absorbed into the systemic circulation [60]. Indole is metabolized by the liver to an IS form, subsequently excreted by the kidneys by organic anion transporter OAT1 and OAT3. On the other hand, indole-3 acetic acid (IAA) is a protein-bound small molecule, also derived from tryptophan metabolism but it is also produced by cells and excreted by tubular secretion mediated by the OAT1 transporter. IS, is an agonist of the transcription factor aryl hydrocarbon receptor (AhR) which regulates the cell response to environmental xenobiotics. IAA may lead to a procoagulant effect, induces endothelial tissue factor expression, and increases the mRNA expression of the enzyme cyclooxygenase-2 (COX-2), which is primarily responsible for the synthesis of inflammatory prostanoides [61,62].

In vitro IAA studies on cultured endothelial cells demonstrated proinflammatory and prooxidant effects, increasing ROS production and the expression of endothelial inflammatory genes: MCP-1, IL-6, IL-8, ICAM-1. Furthermore, AhR activation is associated with oxidative stress, atherogenesis, and vascular inflammation, as well as activation of an inflammatory signaling pathway with p38 MAPK and NF-*κ*B involved in COX-2 upregulation [63].

IS and IAA may contribute to vascular disease progression by AhR activation and increased tissue factor expression in vascular smooth muscle, peripheral blood mononuclear, and endothelial cells [62,64]. The transmembrane glycoprotein TF functions as a high-affinity receptor for FVII and FVIIa factors when TF is exposed to circulation initiates an extrinsic coagulation cascade, so the TF-FVIIa complex is considered the primary activator of the coagulation protease cascade. To allow FVIIa optimal interaction with the substrates FX and FIX, TF is needed to stabilize the catalytic site of FVIIa on a plasma membrane [65].

Extrinsic coagulation can be divided into three phases: TF-FVIIa-dependent initiation, inhibition of the TF-FVIIa complex by TF pathway inhibitor (TFP(I)), and amplification of thrombin generation. In acute atherosclerotic thrombosis, especially acute myocardial infarction, activation of TF is dispensable in the TF-mediated coagulation [57].

Given recent studies on the ability of bacterial metabolites such as TMAO, IS, or IAA to promote the progression or development of cardiovascular diseases, they may have a possible role associated with increased cardiovascular risk.

#### 2.3.2. Effect of Vitamin K-Producing Bacteria

Vitamin K maintains normal blood coagulation. The K-dependent coagulation proteins formed in the liver have coagulant and anticoagulant properties and include factors FII, FVII, FIX, and FX (coagulant role) and proteins C and S (anticoagulant role). It is also involved in the pathway of the glutamate conversion to gamma-carboxyglutamate residues, to form prothrombin. VKAs act as indirect anticoagulants and their principal mechanism of action is based on the inhibition of the vitamin K oxide reductase (VKOR). The gene that codified the VKOR protein, encodes for several isoforms of a protein denominated “vitamin K oxide reductase complex 1” (VKORC1), and its pharmacodynamic is influenced by the vitamin K epoxide reductase (VKORC). VKORC enzyme converts oxidized vitamin K to the reduced active form, and this form is required for the post-translational (gamma) carboxylation of coagulation factors (vitamin K-dependent) [66,67]. Thus, by blocking the reuse of the epoxidemetabolite, VKAs induce, relative, vitamin K deficiency in the cells that synthesize Gla proteins.

There are different forms of vitamin K. Phylloquinone or vitamin K1 is a single compound with a side chain of four isoprenoid residues, three of which are saturated. On the contrary, the menaquinones have side chains of varying length between four and thirteen isoprene residues, most of which are unsaturated [68]. The forms of vitamin K serve as cofactors for the post-translational enzyme γ-glutamate carboxylase. This enzyme converts certain protein-bound glutamate residues into γ-carboxyglutamate or Gla. Seven Glaproteins are involved in blood coagulation (major actors of coagulation cascade) and others are extrahepatically synthesized: osteocalcin (Oc); matrix Gla protein (MGP); Gla-rich protein (GRP); proline-rich Gla proteins (PRGP); 1,2, periostin(isoforms 1–4)/periostin-like-factor (PLF); transmembrane Gla protein (TMG) 3 and 4; and growth arrest specific protein 6 (Gas6) [69]. However, the real physiological importance of these Glas proteins is uncertain.

After intestinal absorption of vitamin K, vitamin K forms are bound to TAG-rich lipoproteins and are transported to the liver and other target tissues. The forms of vitamin K have different pharmacokinetics, with variable plasma half-life times and different tissue distribution. For example, the vitamin K2 MK-7 has a half-life of several days (2–3 days), with higher bioavailability and absorption, whereas vitamins K1 and K2 MK-4 quickly disappear from circulation in the body (half-life of 1 to 2 h) [70].

Diet has an important role in the stability of VKA therapy. For example, patients increasing the intake of vitamin K in the diet may become resistant to the effect of VKAs whereas lower vitamin K intake increases sensitivity to VKAs. Hence, instability in the control of anticoagulation in patients taking VKAs may also be attributable to low vitamin K reserves or poor dietary intake. In clinical trials, vitamin K supplementation is effective for the improvement of the quality of anticoagulation [71].

Why is vitamin K important in patients taking VKA therapy? For patients taking OAC therapy with VKAs, dietary recommendations are based on controlling the intake of vitamin K1 or phylloquinone, present in green leafy vegetables [72,73]. Some forms of vitamin K2 or menaquinones that are present in cheese, natto (Japanese food), and curd, are produced generally by some bacteria genera of the gut microbiota, and 10–25% of total vitamin K intake is derived from menaquinones [74]. Moreover, most anaerobic and aerobic Gram-positive bacteria in the gut use menaquinones in their electron transport pathways. The length of the side chain of menaquinone depends on the growth temperature of a specific bacterial species.

Some examples of menaquinone-producing bacteria are: *Eubacterium lentum* which produces MK-6, *Lactococcus lactis* ssp. *lactis* and spp. *cremoris* that mainly produce MK-8 and MK-9, *Bacteroides fragilis* which produces MK-10, MK-11, and MK-12, whereas some species of the genus *Propionibacterium* mainly produce MK-9 [75].

The absorption routes of menaquinones produced by bacteria are unclear. The absorption of all forms of vitamin K occurs in the small intestine, in a process mediated by bile salts. Nevertheless, most menaquinones are produced in the colon, where the bile salts are absent, suggesting a low absorption of these forms of vitamin K. Some studies in experimental animals [76] and formula milk-fed babies [77] confirm this theory, demonstrating that the absorption of menaquinones intestinally produced is low. Another important factor is that the majority of menaquinones are not bioavailable, as they are inside the membranes of the bacteria that produce them. There may be a role in the coagulation process in patients with prolonged vitamin K deficiency [78].

In some studies [79,80], the effects of menaquinone MK-7 and MK-9 on coagulation parameters have been reported. These studies showed that MK-7 and MK-9 decreased the concentrations of coagulation factors and INR, the effect as an antidote for OAC was stronger for MK-7, being 3 to 4 times more potent for VKAs [81].

Other gut bacteria have been previously identified as menaquinones producers of various chain lengths, such as *Bacteroides ovatus*, *Enterococcus faecalis*, *Escherichia coli*, *Prevotellabuccae*, *Staphylococcus epidermidis*, and *Staphylococcus haemolyticus*. Some strains isolated from feces of exclusively formula-fed infants were reported as vitamin K producers, e.g., *Bacteroides* spp., *Citrobacter freundii*, *Enterobacter agglomerans*, *Enterococcus faecium*, *Serratia marcescens*, *Staphylococcus capitis*, and *Staphylococcus warneri*, these bacterial strains are predominantly facultative anaerobes associated with the young neonatal gut where the environment is more anaerobic [82].

The ability to produce vitamin K allows us to consider the taxonomic composition of the gut microbiota (mediated by vitamin K-producing species) as important factors influencing the anticoagulant effect amongst VKA users.

#### 2.3.3. Structural Modification of the VKAs Molecules by Gut Bacteria

After administration, drug molecules usually undergo chemical modifications and therefore the final metabolites can have different pharmacological properties than the initial molecule [83]. The majority of medications are given orally and they may be exposed to commensal bacteria in the small or large intestines. As for pharmaceutical products, the gut microbiota can transform some drugs by producing metabolites with altered pharmacological properties. Research in this field has determined a complex interaction between drugs-microbiota, either by direct or indirect chemical modification, through the many interactions these organisms have with host cells in this environment [84,85,86].

Previously, some studies have shown interactions between drugs/metabolites and the gut microbiome. For example, many anti-inflammatory agents depend on microbial metabolism to convert inactive precursors (prodrugs) into pharmaceutically active compounds. In the case of sulfasalazine, intestinal microbiota reduces sulfasalazine into sulfapiridine and the active anti-inflammatory agent 5-acetylsalycilic acid (5-ASA) [87] via azoreductase. Some bacterial genera such as *Bacteroides*, *Clostridium*, and *Eubacterium* have been associated with the production of Azo reductases [88,89]. Another case of prodrug activation by gut bacteria includes a sulfoxide reduction in the anti-inflammatory compound sulindac and N oxide reduction of the antidiarrheal drug loperamide [90].

In relation to drugs used in cardiology, the best example is perhaps the *Digitalis purpurea* plant (dedalera), used for years in the treatment of congestive heart failure. The active compound is a cardiac glycoside digoxin [91], which inhibits Na^+^/K^+^ATPases in cardiac myocytes, producing calcium influx and increasing muscle contraction. In studies where antibiotics and digoxin were administered together, dihydrodigoxin production was decreased or eliminated, suggesting a decisive role of the intestinal microbiota in drug inactivation. In 2013, Haiser et al. [92] identified a group of digoxin-inducible genes, only present in *Eggerthellalenta* strains that metabolize digoxin. The cardiac glycoside reductase (cgr) operon from this bacteria, encodes two proteins similar to reductases (Cgr1 and Cgr2) mediating the conversion of digoxin to dihydrodigoxin. These results derived in a dietetic intervention designed for reducing the metabolism of digoxin in vivo.

Another example of microbe-mediated alteration is the uptake and availability of amiodarone (Class III antiarrhythmic). The co-administration of the probiotic bacterium *Escherichia coli* Nissle 1917 serotype O6: K5: H1 (EcN) has been used for the prevention and treatment of some diseases and intestinal disorders such as irritable bowel syndrome, inflammatory bowel disease, and protracted or chronic recurrent diarrhea. EcN can influence the amiodarone pharmacokinetics, increasing its bioavailability. In vivo studies confirmed that EcN increases the bioavailability and absorption of amiodarone [93].

It has been also demonstrated the ability of some bacterial species to chemically modify warfarin [94]. The authors evaluated the drug-metabolizing activity of human gut bacteria in 271 drugs, and they found that about two-thirds of the assayed drugs were metabolized by at least one bacterial strain; this drug-metabolizing activity mediated by the microbiota depends on the drug and its formulation. In the case of warfarin, four bacterial species, such as *Anaerotruncuscolihominis* DSM17241, *Bacteroides vulgatus* ATCC8482, *Collinsellaaerofaciens* ATCC25986, and *Edwardsiellatarda* ATCC23685 were linked to the ability to metabolize this drug. These studies support the theory of possible impact on drug-metabolite exposure, affecting the intestinal and systemic drug metabolism.

### 2.4. Outstanding Questions

With the establishment of the heart-gut axis concept, several studies suggest an important role of the gut microbiota in the pathogenesis of cardiovascular diseases. However, there are limited studies indicating a direct association between gut microbiota and VKAs. Recently, Miamo et al. collected samples from 200 undergoing heart valve replacement patients and the different responses to warfarin anticoagulation therapy were classified as; low, normal, and high responder. The authors observed that the genus *Escherichia-Shigella* and the genus *Enterococcus* were significantly enriched in the low and high response groups, respectively. When the vitamin k content was measured in stool and blood samples, synthesized VK2 and the relative abundance of modules associated with VK biosynthesis and enzymes were higher in the low responder group when compared to normal and high responder [95].

Given this limited data, the possible relationship between gut bacteria and the quality of anticoagulation is still an unexplored field and should be addressed from different perspectives, such as the comparison of microbial profiles in patients with poor and optimal TTR; the identification of microbial signatures using profiled metabolites; and the identification of possible circulating microbial biomarkers to predict a successful VKA treatment.

Overall, our overview suggests a potentially novel causal role of gut microbiota in contributing to the quality of anticoagulation with VKAs users. Herein, we propose that the gut microbiota and microbial products can establish a relationship with the VKA, and we suggested a novel role of the intestinal microbiota as a key factor for interindividual variability in the quality of anticoagulation. It could be assumed that the increase in some specific groups of the gut microbiota may induce metabolic activity that affects the quality of anticoagulation, either by the production of secondary bacterial metabolites, by bacterial groups producing menaquinones, or by structural modification of the anticoagulants drugs. In the future years, new studies focused on the integration of metagenomic and metabolomic analyses may suggest profiles of bacterial signatures or microbial metabolites, to be used as biomarkers to predict the quality of anticoagulation. This could lead to the design of intervention strategies modulating gut microbiota, for example by using probiotic bacteria.

The potential impact of probiotics on the composition of the gut microbiota could be to regulate the synthesis of TMAO precursor molecules in the gut [96]. At the same time, this could decrease the impact on endothelial inflammatory injury, the production of pro-inflammatory cytokines, oxidative stress, and endothelial dysfunction, as well as increased adhesion of monocytes, all associated with TMAO production. A study with potential probiotic bacterial species in mice demonstrated that *Lactobacillus plantarum* ZDY04 significantly reduces serum TMAO and cecal TMA levels by modulating the relative abundance of the families Bacteroidaceae, Erysipelotrichaceae, Lachnospiraceae, and genus *Mucispirillum* [97].

## 3. Conclusions

In anticoagulated patients with VKAs, the quality of anticoagulation control is central to avoiding thromboembolic and bleeding complications. In the search of potential factors affecting the quality of VKAs, gut microbiota, which have shown to play an important role in several cardiovascular diseases, could also have an impact on the response to VKA therapy. This could be the result of an indirect effect of metabolites produced by gut microbiota in the availability of VKAs drugs, an effect of vitamin K-producing bacteria, or by the structural modification of the molecule of VKAs drug. In order to implement predictive or preventive strategies before VKA therapy, there is a need for studies directly investigating the role of gut bacteria and their metabolites on this family of drugs. The use of probiotics could be proposed as an alternative given the modulation of gut microbiota compositions and their metabolic pathways. The main strategy is based on modulating the intestinal ecosystem using bacterial species with the ability to compete for the colonization of the intestinal epithelium or with antimicrobial or antagonist activity against metabolite-producing bacteria such as TMAO, vitamin K-producing bacteria, or bacterial with the ability to structurally modify the molecules of VKAs.

## Figures and Tables

**Table 1:** Factors affecting the quality of vitamin K antagonist therapy.

**Co-Medication**
Antiplatelet drugs
Drugs affecting pharmacokinetics or pharmacodynamics of VKAs
*Non-steroidal* anti-inflammatory drugs
**Comorbidities**
Cancer
Congestive heart failure
Liver diseases
History of atherosclerotic stroke
History of major bleeding
Uncontrolled hypertension
**Genetic Factors**
Mutation in factor IX propeptide (low factor IX levels)
Polymorphisms of VKORC1 and CYP2C9
**Natural Conditions**
Advanced age
Female sex
**Personal Characteristics/Life Habits**
Absence of familiar or social support
Alcohol abuse
Insufficient information and education to the treatment
Nutritional supplements and herbal products
Poor compliance
Poor dietary intake of vitamin K
Tendency to falls

VKAs: vitamin K antagonists.

## Data Availability

Not applicable.

## Abbreviations

APS	antiphospholipid syndrome
COX-2	cyclooxygenase-2
DOACs	direct-acting oral anticoagulants
Gas6	growth arrest specific protein 6
GBB	γ-butyrobetaine
GM	gut microbiota
GP	ganglionated plexi
GRP	gla-rich protein
HMGB1	high mobility group box 1
IAA	indole-3 acetic acid
INR	international normalized ratio
IS	indoxyl Sulfate
MGP	matrix Gla protein
NF-κB	nuclear factor-κB
OAC	oral anticoagulation
OACs	oral anticoagulants
Oc	osteocalcin
PLF	periostin-like-factor
PRGP	proline-richGla proteins
TMA	trimethylamine
TMAO	trimethylamine N-Oxide
TMG	transmembrane Gla protein
TNF-α	tumor necrosis factor-alpha
TTR	therapeutic range
VKAs	vitamin K antagonists
VKORC	vitamin K epoxide reductase
VKORC1	vitamin K oxide reductasecomplex 1
VTE	venous thromboembolism
